# ﻿Five new species of *Bryaxis* Kugelann (Coleoptera, Staphylinidae, Pselaphinae) from Korea and a nomenclatural note on *Bryaxismahunkai* Löbl

**DOI:** 10.3897/zookeys.1182.97346

**Published:** 2023-10-13

**Authors:** Yeon-Jae Choi, Sun-Jae Park, Seung-Gyu Lee, Jong-Seok Park

**Affiliations:** 1 Department of Biological Sciences and Biotechnology, Chungbuk National University, Cheongju 28644, Republic of Korea; 2 Climate Change and Environmental Biology Research Division, National Institute of Biological Resources, Incheon 22689, Republic of Korea; 3 Biodiversity Education Division, National Institute of Biological Resources, Incheon 22689, Republic of Korea

**Keywords:** *B.fabaiformis* sp. nov., *B.girinensis* sp. nov., *B.grandinodus* sp. nov., *B.nemorosus* sp. nov., *B.uljinensis* sp. nov., diversity, morphology, new synonym, Palearctic, taxonomy

## Abstract

The genus *Bryaxis* Kugelann (Goniaceritae: Bythinini) is the most species-rich genus of the subfamily Pselaphinae and is mainly distributed in the Palearctic region. Although previous studies have documented 14 species in the Korean Peninsula, the true diversity, ecology, and immature stages of the genus are still inadequately known. In this study, five new Korean species are described: *B.grandinodus***sp. nov.**, *B.uljinensis***sp. nov.**, *B.fabaiformis***sp. nov.**, *B.girinensis***sp. nov.**, and *B.nemorosus***sp. nov.** Illustrations of the habitus and other morphological details, and a distribution map are provided. In addition, *Bryaxisleechanyoungi* Nomura & Lee, 1993 is proposed as a new synonym of *B.mahunkai* Löbl, 1975 based on the original description and illustrations of diagnostic characters.

## ﻿Introduction

The genus *Bryaxis* Kugelann, 1794 is the most species-rich pselaphine genus, containing 385 species and 40 subspecies. Except for one adventive species recorded from North America (Chandler 2022) most species of the genus are distributed in the Palearctic and Oriental regions (Newton 2022; Yin 2023). In Northeast Asia, 36, 10, 18, and 19 species are recorded in Japan, the Russian Far East, China, and Taiwan, respectively (Schülke and Smetana 2015; Taru and Nomura 2021; Yin 2023). In Korea, *Bryaxis* comprises 14 species, 11 of which are endemic (Schülke and Smetana 2015; Ahn et al. 2017). Löbl (1974) first recorded this genus in Korea by describing two species, *B.pawlowskii* Löbl and *B.validicornides* Löbl. Nomura and Lee (1992, 1993) revised the Korean *Bryaxis* and described eight species, one of which was later synonymized (*B.coreanus* Nomura & Lee, 1992 with *B.koltzei* (Reitter, 1887); Nomura 1995). Members of Korean *Bryaxis* can be identified by the swollen antennal scape or pedicel with glandular nodule in males (Nomura and Lee 1992). All type specimens were collected from forest leaf litter.

Herein we describe five new species by providing illustrations of the habitus and diagnostic characters of each species and a distribution map. Moreover, we found a taxonomic problem regarding *Bryaxisleechanyoungi* Nomura & Lee, 1993, which is synonymized with *B.mahunkai* Löbl, 1975 in the present study.

## ﻿Material and method

Eighty-seven specimens from Chungbuk National University Insect Collection (**CBNUIC**, Cheongju, Republic of Korea) and one specimen from Chungnam National University Insect Collection (**CNUIC**, Daejeon, Republic of Korea) were examined. The holotypes of all species described herein are deposited in the National Institute of Biological Resources (**NIBR**, Incheon, Republic of Korea). Depositions of paratypes and vouchers are indicated parenthetically. At least one specimen of each species was dissected to study the male genitalia and details of other characters. Terminology and nomenclature used follow Chandler (2001) for external characters and Lawrence et al. (2011) for genital characters. Numbering of abdominal sclerites indicate morphological segments. Specimen label data for the holotypes are transcribed verbatim. Data for other specimens are standardized for consistency. Specimens were observed using a Leica M80 and DM1000 LED optical microscope. Images were generated using Sony ILCE-7RM3 mirrorless camera and stacked with Zerene Stacker v. 1.04. The map of Korea was created using the Natural Earth quick start for QGIS v. 3 and open source QGIS v. 3.30.2. For comparison, localities of three dominant species in Korea, *Bryaxismahunkai* Löbl, *B.koltzei* (Reitter) and *B.kimjongkuki* Nomura & Lee, were also marked.

## ﻿Results

### ﻿Subfamily Pselaphinae Latreille, 1802

**Supertribe Goniaceritae Reitter, 1882**

**Tribe Bythinini Raffray, 1890**

#### ﻿Genus Bryaxis

Taxon classificationAnimaliaColeopteraStaphylinidae

Kugelann, 1794

90C5965E-45F9-53D2-A81A-6010E3847D19

##### Type species.

*Pselaphusbulbifer* Reichenbach, 1816.

#### ﻿ Bryaxis grandinodus

Taxon classificationAnimaliaColeopteraStaphylinidae

Choi, Park, Lee & Park sp. nov.

1A296044-ED59-5F88-B5E6-701BCF2B01F2

https://zoobank.org/296C6B58-60CE-41D9-BD87-AAC45B5AC0C5

Figs 1 , 2A, C

##### Type materials

**(*N* = 11, 6**♂♂, **5**♀♀) **. *Holotype male*.** “Korea: Jeonnam Prov. Dangsan-ri, Gyegok-myeon, Haenam-gun, 18 May 2019, 34°40'53.0"N, 126°38'56.3"E, 211 m, sifting leaf litter & deadwood debris, J.-S. Park, M.-H. Song” (NIBR). ***Paratypes*.** 2♂♂, 2♀♀ (CBNUIC, 1♂, 1♀ slide mounted, 1♂, 1♀ dried). “Korea: Jeonbuk Prov. Sinsi island. Sinsido-gil, Okdo-myeon, Gunsan-si, 4 Jul 2022, 35°49'12.2"N, 126°27'35.1"E, 36 m, sifting leaf & soil litter, M.-H. Song, U.-J. Byeon, J.-W. Kang, T.-Y. Jang”. 2♂♂ (CBNUIC, dried) “Korea: Jeonbuk Prov. Seonyu island. Seonyubuk-gil, Okdo-myeon, Gunsan-si, 16 Jun 2021, 35°48'36.5"N, 126°24'57.4"E, 25 m, sifting leaf, soil litter & fungi, J.-W. Kang, J.-I. Shin”. 1♂, 3♀♀ (CBNUIC, DNA grade). “Korea: Jeonbuk Prov. Seonyu island. 5-1, Seonyunam-gil, Okdo-myeon, Gunsan-si, 4 Jul 2022, 35°48'24.7"N, 126°24'40.3"E, 9 m, sifting leaf & soil litter, M.-H. Song, U.-J. Byeon, J.-W. Kang, T.-Y. Jang”.

##### Diagnosis.

**Figure 1.**
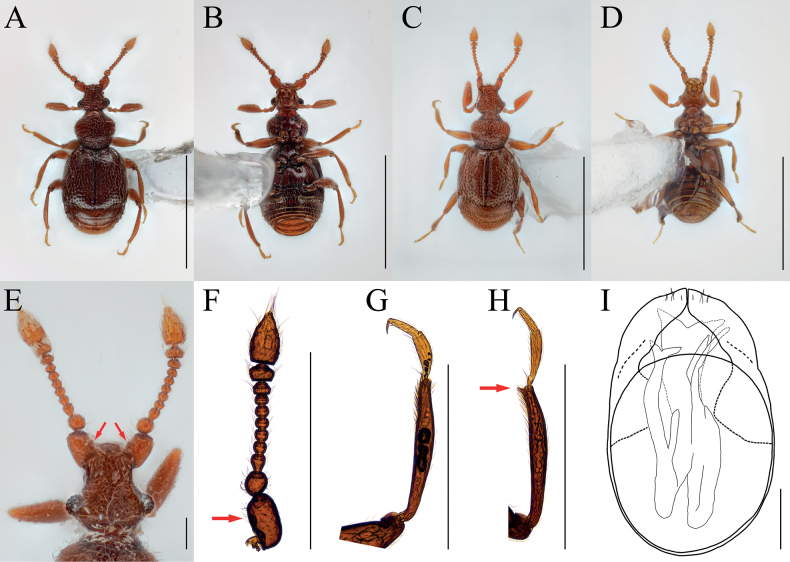
*Bryaxisgrandinodus* Choi, Park, Lee & Park, sp. nov., male (**A, B, E–I**), female (**C, D**). **A, C** dorsal habitus **B, D** ventral habitus **E** head **F** antenna **G** fore leg **H** hind leg **I** aedeagus. Scale bars: 1 mm (**A–D**); 0.1 mm (**E, I**); 0.5 mm (**F–H**).

**Figure 2.**
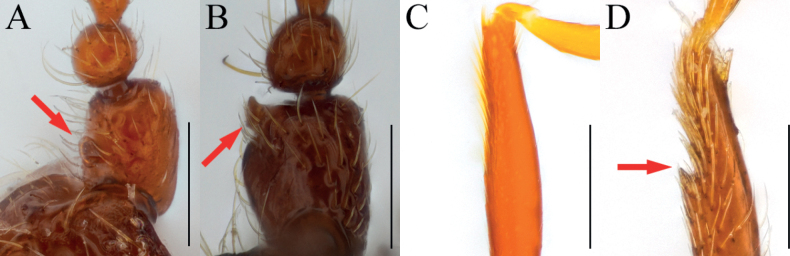
Diagnostic characters of *Bryaxisgrandinodus* sp. nov. (**A, C**) and *B.koltzei* (Reitter) (**B, D**). **A, B** antennal scapes and pedicels **C, D** protibiae. Scale bars: 0.1 mm.

Antennal scapes robust, with bowl-like glandular nodule on inner margin (Figs 1E, F, 2A, arrows), 2.45 times as long as pedicels; endophallus of male genitalia with three bifid struts, joined at base (Fig. 1I).

##### Description.

Body reddish brown, antennae, maxillary palpi, and tarsi slightly lighter, length 1.2–1.32 mm, maximum width 0.51–0.58 mm (Fig. 1A–D). Setae on body yellowish, long. Head 0.92 times as long as wide (Fig. 1E). Frons with U-shaped impression between antennal tubercles; frontal foveae absent; frontal rostrum distinct anteriorly. Vertex slightly convex; longitudinal carina weak; vertexal foveae large. Eyes as long as tempora, with 23–26 facets. Maxillary palpi moderately developed; palpomeres II–III with tubercles; palpomere IV 0.23 mm long and about 3.15 times as long as wide, subcylindrical pseudosegment at apex. Antennae about 0.54 mm long; pedicels subglobose with long setae, 0.89 times as long as wide; antennomere III 1.12 times as long as wide; IV–VIII subequal in length; IX–X transverse, IX 0.64 times as long as wide and X 0.63 times as long as wide; XI largest, pointed at apex, 1.67 times as long as wide (Fig. 1F). Pronotum 0.84 times as long as wide and widest at basal 2/3, lateral antebasal foveae connected by antebasal sulcus. Elytra slightly convex, 0.89 times as long as wide and widest at basal 1/4, each elytron with two basal foveae and subhumeral fovea. Legs slender; protibiae without spine (Figs 1G, 2C); metatibiae with spine at apex (Fig. 1H, arrow). Aedeagus large, 0.37 mm long and 1.69 times as long as wide; penis bulbous and dorsal diaphragm ovoid; parameres short and symmetrical, apices almost encountered, one robust seta and three fine setae on each apex; endophallus divided into three large struts, left dorsal strut branched at basal 1/3 and bifid at apex, right dorsal strut weakly branched basally and bifid dorso-ventrally at apex, ventral strut robust and bifid at apex (Fig. 1I).

***Sexual dimorphism*.** Female eyes shorter than tempora, composed of 11 facets; antennal scapes subcylindrical, without modification; metatibial spines absent.

##### Remarks.

Adults of this species are very similar to *Bryaxiskoltzei* (Reitter, 1887) in the general body characters, but can be distinguished by the shape of the antennal scapes and its glandular nodule (Fig. 2A, arrow) and the spineless protibiae in the male (Fig. 2C).

##### Comments.

The localities of *B.grandinodus* sp. nov. probably overlap with those of *B.koltzei* (Reitter) given that the latter are distributed across the entire country (Fig. 11).

##### Etymology.

The specific epithet is a combination of the Latin words *grandis* (“large”, masculine) and *nodus* (“knob”, masculine) and refers to the shape of the glandular nodules on the male antennal scapes.

##### Habitat.

The holotype was collected by sifting leaf litter in mixed forest. Paratypes were collected by sifting leaf litter and soil.

##### Distribution.

Korea (Haenam-gun, Jeollanam-do; Gunsan-si, Jeollabuk-do).

#### ﻿ Bryaxis uljinensis

Taxon classificationAnimaliaColeopteraStaphylinidae

Choi, Park, Lee & Park sp. nov.

CD7668F6-5DCD-54EB-8AAD-2B5830003613

https://zoobank.org/CF039CE1-2159-4BF5-8E6A-5B9508CBEAC5

Figs 3 , 4A, C, E

##### Type materials

**(*N* = 7, 4**♂♂, **3**♀♀) **. *Holotype male*.** “Korea: Gyeongbuk Prov. Onjeong-myeon, Uljin-gun, 8 Jun 2019, 36°43'23.0"N, 129°20'16.0"E, 180 m, sifting leaf litter near stream, J.-S. Park” (NIBR). ***Paratypes*.** 2♂♂ (CBNUIC, 1♂ slide mounted, 1♂ dried). “Korea: Gangwon Prov. Gujeol-ri, Yeoryang-myeon, Jeongseon-gun, 24 Apr 2020, 37°31'08.7"N, 128°46'42.8"E, 591 m, sifting leaf & soil litter, U.-J. Byeon, T.-Y. Jang”. 1♂, 3♀♀ (CBNUIC, dried). “Korea: Gangwon Prov. Gujeol-ri, Yeoryang-myeon, Jeongseon-gun, 24 Apr 2020, 37°30'57.6"N, 128°45'18.8"E, 510 m, sifting ant colony, leaf & soil litter, Y.-J. Choi, U.-J. Byeon”.

##### Diagnosis.

**Figure 3.**
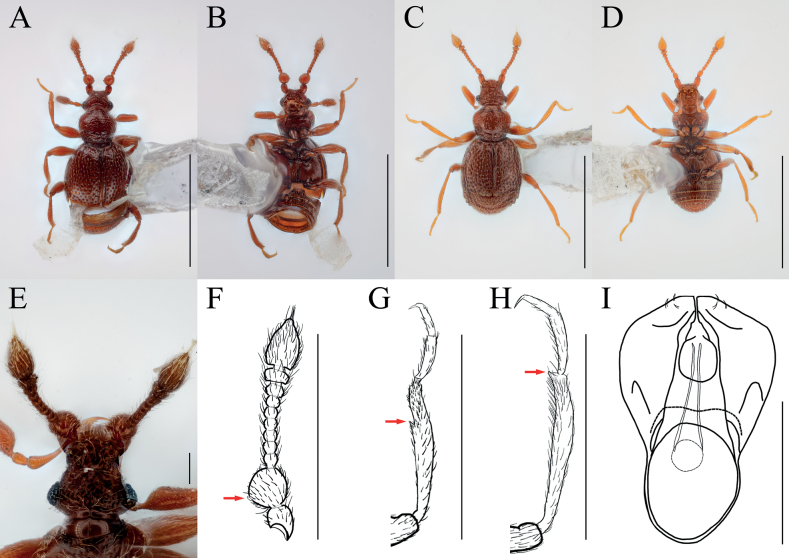
*Bryaxisuljinensis* Choi, Park, Lee & Park, sp. nov., male (**A, B, E–I**), female (**C, D**). **A, C** dorsal habitus **B, D** ventral habitus **E** head **F** antenna **G** fore leg **H** hind leg **I** aedeagus. Scale bars: 1 mm (**A–D**); 0.1 mm (**E, I**); 0.5 mm (**F–H**).

**Figure 4.**
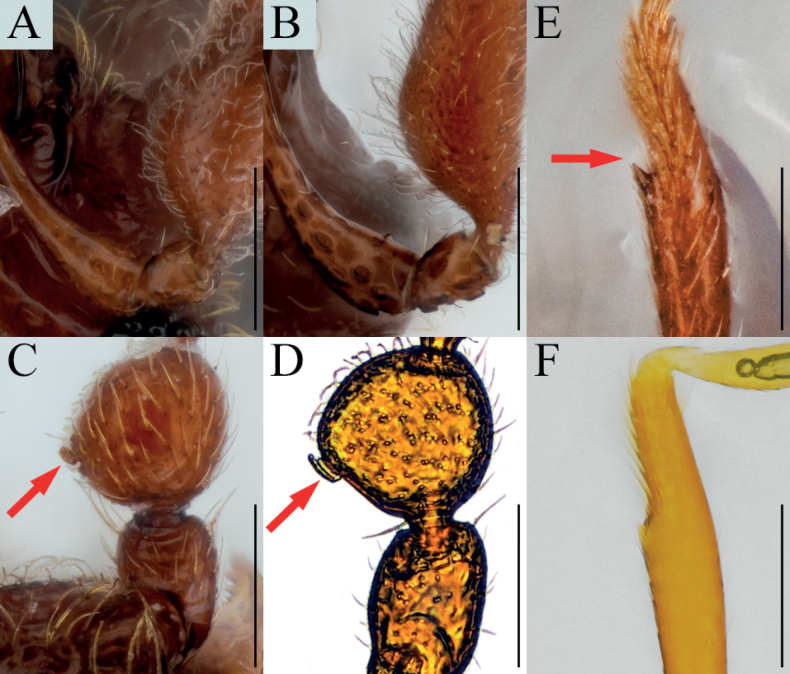
Diagnostic characters of *Bryaxisuljinensis* sp. nov. (**A, C, E**) and *B.mahunkai* Löbl (**B, D, F**). **A, B** maxillary palpi **C, D** antennal scapes and pedicels **E, F** protibiae. Scale bars: 0.1 mm.

Antennal pedicel strongly swollen, subglobose with subcylindrical glandular nodule on basal 1/3 of inner margin (Figs 3F, 4C, arrows); protibiae with spine on internal side of widest (Figs 3G, 4E, arrows); parameres of male genitalia robust and fan-shaped, bearing three setae on each (Fig. 3I).

##### Description.

Body reddish brown, antennae, maxillary palpi, legs slightly lighter than body, length 1.25–1.31 mm, maximum width 0.53–0.60 mm (Fig. 3A–D). Setae on body yellowish, and short. Head 0.98 times as long as wide (Fig. 3E). Frons with small U-shaped impression between antennal tubercles; frontal foveae absent; frontal rostrum slightly distinct anteriorly. Vertex convex; longitudinal carina present; vertexal foveae small. Eyes large, longer than twice that of tempora, composed of 32–34 facets. Maxillary palpi moderately developed; palpomeres II–III smooth; palpomere IV 0.22 mm long and about 3.16 times as long as wide, subcylindrical pseudosegment at apex. Antennae about 0.53 mm long; scapes short without modification, 0.92 times as long as pedicels; pedicels long as wide; antennomere III–VIII subequal in length; IX 0.63 times as long as wide; X transverse, 0.61 times as long as wide; XI largest, pointed at apex, 1.61 times as long as wide (Fig. 3F). Pronotum 0.88 times as long as wide and widest at basal 3/5, lateral antebasal foveae connected by antebasal sulcus. Elytra convex, 0.85 times as long as wide and widest at basal 1/3, each elytron with two basal foveae and subhumeral fovea. Legs robust; internal spine on widest of protibiae (Fig. 3G, arrow); metatibiae with spine on apical (Fig. 3H, arrow). Aedeagus small, 0.27 mm long and 1.61 times as long as wide; penis small fusiform and dorsal diaphragm bulbous; parameres symmetrical; endophallus composed with two convergent, slender struts (Fig. 3I).

***Sexual dimorphism*.** Female eyes slightly longer than tempora, composed of 15 facets; antennal pedicels simple; protibial spines and metatibial spines absent.

##### Remarks.

Adults of this species are very similar to *Bryaxismahunkai* Löbl, 1975 in having strongly swollen antennal pedicels (Fig. 4C, D). However, they can be distinguished by smooth maxillary palpomere II–III (Fig. 4A), apically symmetrical antennal scapes, pedicels less swollen apically and bearing smaller glandular nodules (Fig. 4C, arrow), fore legs with a tibial spine (Fig. 4E, arrow), and parameres wider than the penis (aedeagus in *B.mahunkai* as wide as penis; Fig. 10C, D).

##### Comments.

The localities of *B.uljinensis* sp. nov. probably overlap with those of *B.mahunkai* Löbl given that the latter are nationally distributed (Fig. 11).

##### Etymology.

This species is named after the type locality, Uljin-gun.

##### Habitat.

The holotype was collected by sifting leaf litter in mixed forest. Paratypes were collected by sifting leaf litter, soil, and an ant colony.

##### Distribution.

Korea (Uljin-gun, Gyeongsangbuk-do; Jeongseon-gun, Gangwon-do).

#### ﻿ Bryaxis fabaiformis

Taxon classificationAnimaliaColeopteraStaphylinidae

Choi, Park, Lee & Park sp. nov.

B506A12E-CEE7-5E26-B352-AF3D4617C6BE

https://zoobank.org/1110CCA6-D13F-45B9-A9EF-49B993E8DF44

Figs 5 , 6A, C, E

##### Type materials

**(*N* = 4, 3**♂♂, **1**♀) **. *Holotype male*.** “Korea: Gangwon Prov. Gujeol-ri, Yeoryang-myeon, Jeongseon-gun, 24 Apr 2020, 37°31'08.3"N, 128°46'43.0"E, 552 m, sifting soil & leaf litter, U.-J. Byeon, T.-Y. Jang” (NIBR). ***Paratypes*.** (CBNUIC, 1♂ slide mounted, 1♂, 1♀ dried). “Korea: Gangwon Prov. Gujeol-ri, Yeoryang-myeon, Jeongseon-gun, 24 Apr 2020, 37°31'08.3"N, 128°46'43.0"E, 552 m, sifting soil & leaf litter, U.-J. Byeon, T.-Y. Jang”.

##### Diagnosis.

**Figure 5.**
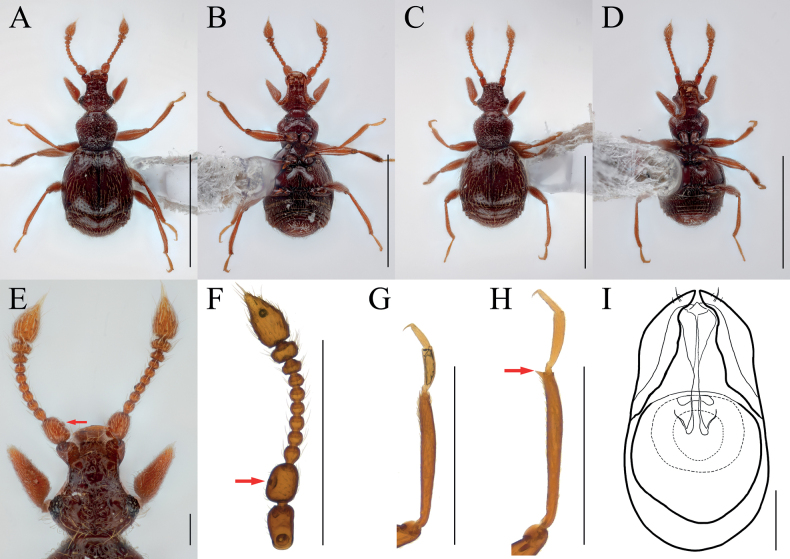
*Bryaxisfabaiformis* Choi, Park, Lee & Park, sp. nov. male (**A, B, E–I**), female (**C, D**). **A, C** dorsal habitus **B, D** ventral habitus **E** head **F** antenna **G** fore leg **H** hind leg **I** aedeagus. Scale bars: 1 mm (**A–D**); 0.1 mm (**E, I**); 0.5 mm (**F–H**).

**Figure 6.**
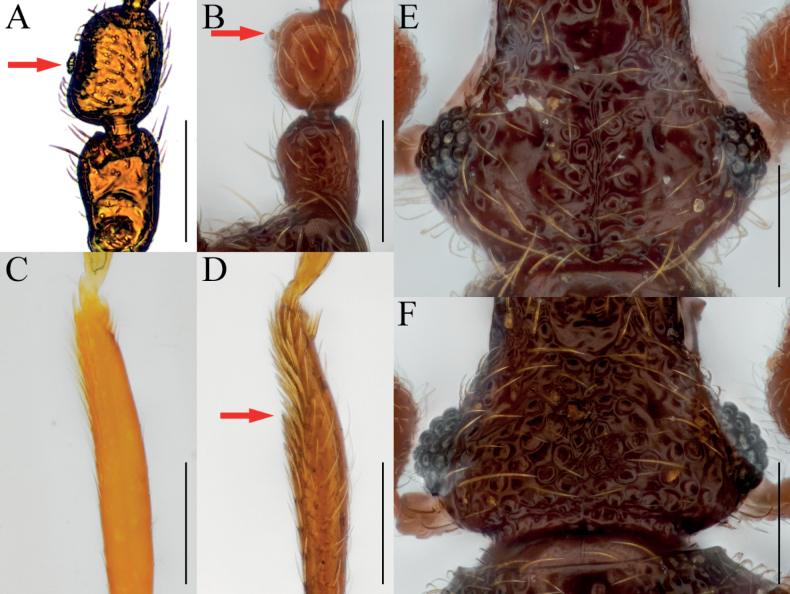
Diagnostic characters of *Bryaxisfabaiformis* sp. nov. (**A, C, E**) and *B.kimjongkuki* Nomura & Lee (**B, D, F**). **A, B** antennal scapes and pedicels **C, D** protibiae **E, F** heads. Scale bars: 0.1 mm.

Enlarged fabiform antennal pedicels with subcylindrical glandular nodule on inner margin in male (Figs 5E, F, 6A, arrows).

##### Description.

Body reddish brown, antennae, maxillary palpi, legs slightly lighter than body, length 1.29–1.41 mm, maximum width 0.60–0.64 mm (Fig. 5A–D). Setae on body golden and long. Head 0.84–1.00 times as long as wide (Fig. 5F). Frons with U-shaped impression between antennal tubercles; frontal foveae absent; frontal rostrum distinct anteriorly. Vertex weakly convex; longitudinal carina distinct; vertexal foveae enlarged. Eyes large with 31–32 facets. Maxillary palpi moderately developed; palpomeres II–III with sparse tubercles; palpomere IV 0.25–0.29 mm long and about 2.92–3.44 times as long as wide, subcylindrical pseudosegment at apex. Antennae about 0.58–0.61 mm long; scapes short, without modification, 0.73–1.11 times as long as pedicels; pedicels 1.02–1.19 times as long as wide; antennomere III 1.18–1.23 times as long as wide; IV–VIII subequal in length; IX–X transverse, IX 0.66–0.69 times as long as wide and X 0.62–0.63 times as long as wide; XI largest, pointed at apex, 1.65–1.75 times as long as wide (Fig. 5F). Pronotum 0.84–0.89 times as long as wide and widest at basal 3/5, lateral antebasal foveae connected by antebasal sulcus. Elytra convex, 0.85–0.94 times as long as wide and widest at basal 1/3, each elytron with two basal foveae and subhumeral fovea. Legs slender; protibiae without spine (Fig. 5G); metatibiae with spine at apex (Fig. 5H, arrow). Aedeagus robust, 0.43 mm long and 1.79 times as long as wide; penis bulbous and dorsal diaphragm circular; parameres small and symmetrical, each with three setae; endophallus comprising two symmetrical struts, each broadened basally and apically, and shortly branched basally (Fig. 5I).

***Sexual dimorphism*.** Female eyes slightly shorter than tempora, comprising 9 facets; antennal pedicels without modification; metatibial spines absent.

##### Remarks.

Adults of this species are similar to those of *Bryaxiskimjongkuki* Nomura & Lee, 1993 in having the maxillary palpomere II–III with tubercles and asymmetrical antennal scapes. However, they can be recognized by having a rounded tempora as long as the eyes (Fig. 6E), a glandular nodule situated at the mid-level of the antennal pedicels (Fig. 6A, arrow), and protibiae without a spine (Fig. 6C).

##### Comments.

The localities of *B.fabaiformis* sp. nov. probably overlap with those of *B.kimjongkuki* Nomura & Lee given that the latter species was abundantly collected near the type localities of the former (Fig. 11).

##### Etymology.

The specific epithet is a combination of the Latin words *faba* (“bean”, feminine) and -*formis* (“having the form of”, masculine/feminine) and refers to the shape of antennal pedicels in the male.

##### Habitat.

Specimens of this species were collected by sifting soil and leaf litter in mixed forest.

##### Distribution.

Korea (Jeongseon-gun, Gangwon-do).

#### ﻿ Bryaxis girinensis

Taxon classificationAnimaliaColeopteraStaphylinidae

Choi, Park, Lee & Park sp. nov.

2189D25A-258A-5B93-ADC8-71FB7CDDE285

https://zoobank.org/DB5551C4-3A0F-4F13-8D83-87BFBAA8741A

Figs 7 , 9A, C, E

##### Type material

**(*N* = 1, 1**♂) **. *Holotype male*.** “Korea: Gangwon Prov. Bangdong-ri, Girin-myeon, Inje-gun, 23 Jun 2009, sifting flood debris, T.-K. Kim, CNUIC” (NIBR).

##### Diagnosis.

**Figure 7.**
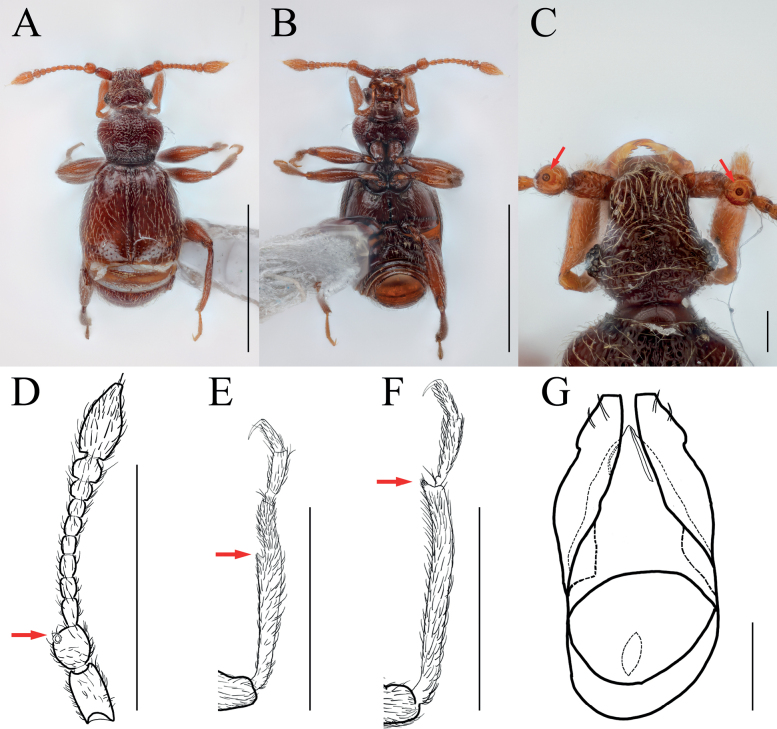
*Bryaxisgirinensis* Choi, Park, Lee & Park, sp. nov. **A** dorsal habitus **B** ventral habitus **C** head **D** antenna **E** fore leg **F** hind leg **G** aedeagus. Scale bars: 1 mm (**A, B**); 0.1 mm (**C, G**); 0.5 mm (**D–F**).

Antennal pedicels less enlarged subglobose, with dorsolateral glandular nodule on subapical (Fig. 7C, D, arrows); protibiae with spine on internal side at widest point (Figs 7E, 9C, arrows); parameres of male genitalia with depression on lateral margin and three setae on apical (Fig. 7G).

##### Description.

Body reddish brown, antennae, maxillary palpi, and tarsi slightly lighter, length 1.62 mm, maximum width 0.70 mm (Fig. 7A, B). Setae on body yellowish, long and dense. Head long as wide (Fig. 7C). Frons with U-shaped impression between antennal tubercles; frontal foveae absent; frontal rostrum distinct anteriorly. Vertex slightly convex; longitudinal carina present; vertexal foveae small. Eyes large with 34 facets. Maxillary palpi moderately developed; palpomeres II–III with dense tubercles; palpomere IV 0.25 mm long and about 3.30 times as long as wide, subcylindrical pseudosegment at apex. Antennae about 0.54 mm long; scapes subcylindrical, without modification, 1.83 times as long as pedicels; pedicels long as wide; antennomere III 1.70 times as long as wide; IV–VIII subequal in length; IX 0.89 times as long as wide; X 0.78 times as long as wide; XI largest, pointed at apex, 1.86 times as long as wide (Fig. 7D). Pronotum 0.79 times as long as wide and widest at basal 3/5, lateral antebasal foveae connected by antebasal sulcus. Elytra slightly convex, 0.98 times as long as wide and widest at basal 1/3, each elytron with two basal foveae and subhumeral fovea. Legs robust; metatibiae with spine at apex (Fig. 7E, arrow). Aedeagus large, 0.36 mm long and 2.05 times as long as wide; penis fusiform and dorsal diaphragm transversely ovoid; parameres short and symmetrical, apices truncated; endophallus composed of two fine struts, asymmetrical (Fig. 7G).

***Sexual dimorphism*.** Unknown.

##### Remarks.

**Figure 8.**
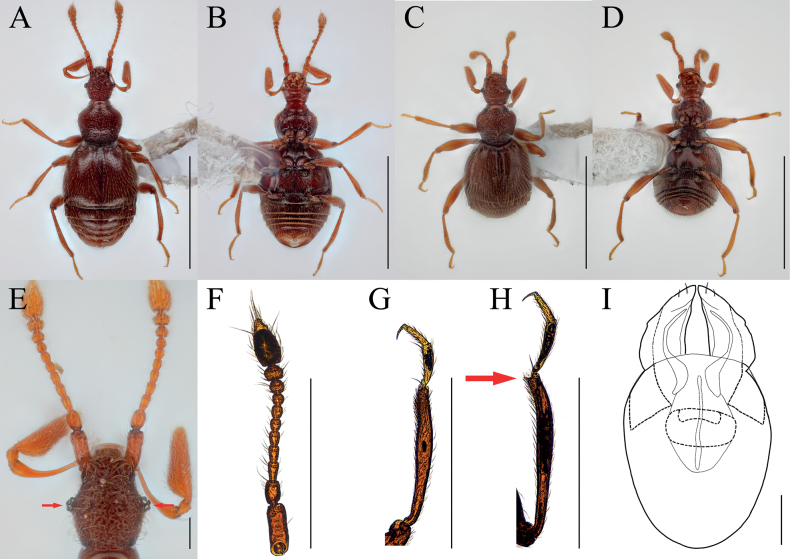
*Bryaxisnemorosus* Choi, Park, Lee & Park, sp. nov. male (**A, B, E–I**), female (**C, D**). **A, C** dorsal habitus **B, D** ventral habitus **E** head **F** antenna **G** fore leg **H** hind leg **I** aedeagus. Scale bars: 1 mm (**A–D**); 0.1 mm (**E, I**); 0.5 mm (**F–H**).

**Figure 9.**
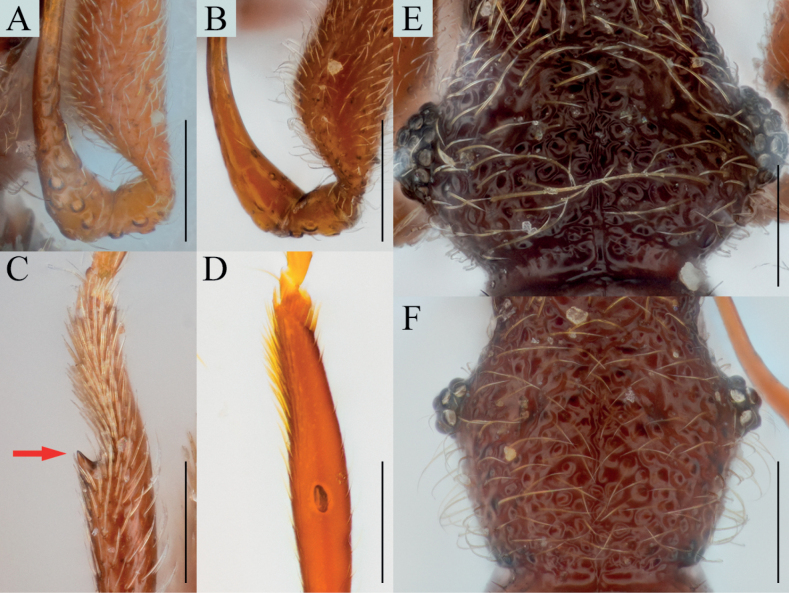
Diagnostic characters of *Bryaxisgirinensis* sp. nov. (**A, C, E**) and *B.nemorosus* sp. nov. (**B, D, F**). **A, B** maxillary palpi **C, D** protibiae **E, F** heads. Scale bars: 0.1 mm.

The adult of this species is similar to *Bryaxisnemorosus* Choi, Park, Lee & Park sp. nov. in the shape of antennomeres IV–XI (Figs 7D, 8F). However, it can be distinguished by the robust setae on the body (Fig. 7A), large eyes as long as the tempora (Fig. 9E), a strongly tuberculate maxillary palpomere II (Fig. 9A), protibiae with a spine at the widest point (Fig. 9C, arrow), and a simple endophallus of the male genitalia (Fig. 7G).

##### Etymology.

This species is named after the type locality, Girin-myeon, Inje-gun.

##### Habitat.

The holotype was collected by sifting flood debris in mixed forest.

##### Distribution.

Korea (Inje-gun, Gangwon-do).

#### ﻿ Bryaxis nemorosus

Taxon classificationAnimaliaColeopteraStaphylinidae

Choi, Park, Lee & Park sp. nov.

C69925DF-8958-5287-953C-D03ACA412693

https://zoobank.org/812B1845-BAEB-4E11-A8BD-7F51B922433F

Figs 8 , 9B, D, F

##### Type materials

**(*N* = 5, 4**♂♂, **1**♀) **. *Holotype male*.** “Korea: Jeonnam Prov. Mt. Doksil, Gageo island. Gageodo-gil, Heuksan-myeon, Sinan-gun, 13 Jul 2021, 34°05'06.1"N, 125°06'17.4"E, 468 m, sifting leaf & soil litter, J.-W. Seo” (NIBR). ***Paratype*.** 1♂ (CBNUIC, slide mounted). “Korea: Jeonnam Prov. Mt. Doksil, Gageo island. Gageodo-gil, Heuksan-myeon, Sinan-gun, 13 Jul 2021, 34°05'06.1"N, 125°06'17.4"E, 468 m, sifting leaf & soil litter, J.-W. Seo”. 1♂ (CBNUIC, dried). “Korea: Jeonnam Prov. Mt. Doksil, Gageo island. Gageodo-gil, Heuksan-myeon, Sinan-gun, 8 Jul 2020, 34°05'35.0"N, 125°06'25.0"E, 590 m, sifting leaf & soil litter, T.-Y. Jang”. 1♂ (CBNUIC, dried). “Korea: Jeonnam Prov. Mt. Doksil, Gageo island. Gageodo-gil, Heuksan-myeon, Sinan-gun, 8 Jul 2020, 34°04'40.0"N, 125°06'23.0"E, 540 m, sifting leaf & soil litter, U.-J. Byeon, T.-Y. Jang”. 1♀ (CBNUIC, dried). “Korea: Jeonnam Prov. Mt. Doksil, Gageo island. Gageodo-gil, Heuksan-myeon, Sinan-gun, 7 Jul 2020, 34°04'34.7"N, 125°06'28.8"E, 534 m, sifting leaf & soil litter, T.-Y. Jang”.

##### Diagnosis.

Elongated head with small eyes situated on mid-length of head (Fig. 8E, arrow).

##### Description.

Body reddish brown, antennae, maxillary palpi, legs slightly lighter than body, length 1.57–1.58 mm, maximum width 0.61–0.67 mm (Fig. 8A–D). Setae on body yellowish. Head 1.15 times as long as wide (Fig. 8E). Frons with U-shaped impression between antennal tubercles; frontal foveae absent; frontal rostrum distinct anteriorly. Vertex weakly convex; longitudinal carina absent; vertexal foveae small. Eyes reduced with 9 facets. Maxillary palpi moderately developed; palpomeres II smooth; III with tubercles; palpomere IV 0.24–0.28 mm long and about 3.06–3.24 times as long as wide, subcylindrical pseudosegment at apex. Antennae about 0.61–0.68 mm long; scapes subcylindrical and elongated, 2–2.18 times as long as pedicels, without modification; pedicels subcylindrical, 1.38–1.40 times as long as wide; antennomere III 1.67–1.72 times as long as wide; IV–VII subequal in length; VIII subglobose as long as wide; IX 0.82–0.86 times as long as wide; X transverse, 0.64–0.65; XI largest, pointed at apex, 1.76–1.84 times as long as wide (Fig. 8F). Pronotum 0.85–0.89 times as long as wide and widest at basal 2/3, lateral antebasal foveae connected by antebasal sulcus. Elytra slightly convex, 0.87 times as long as wide and widest at basal 1/3, each elytron with two basal foveae and subhumeral fovea. Legs slender; protibiae without spine (Fig. 8G); metatibiae with spine on apical (Fig. 8H, arrow). Aedeagus robust, 0.53 mm long and 1.79 times as long as wide; penis bulbous and dorsal diaphragm small, transversely ovoid; parameres symmetrical, each bearing two setae; endophallus comprising simple strut basally and two symmetrical struts curved along with parameres, thickened basally (Fig. 8I).

***Sexual dimorphism*.** Female metatibial spines absent.

##### Remarks.

Adults of this species are similar to that of *Bryaxisgirinensis* Choi, Park, Lee & Park sp. nov. in the shape of antennomeres IV–XI (Figs 7D, 8F). However, they can be distinguished by having angular tempora much longer than the eyes (Fig. 9F), smooth maxillary palpomere II (Fig. 9B), unadorned antennal scapes and pedicels (Fig. 8F), slender protibiae without a spine (Fig. 9D), and an endophallus composed of three long struts (Fig. 8I).

##### Etymology.

The specific epithet is the Latin word *nemorosus* (“wooded, shady”, masculine) that refers to habitat where the types collected.

##### Habitat.

Specimens of this species were collected by sifting leaf and soil litter in wet forest with dense canopy, which is located on an island.

##### Distribution.

Korea (Gageo island, Sinan-gun, Jeollanam-do).

#### ﻿ Bryaxis mahunkai

Taxon classificationAnimaliaColeopteraStaphylinidae

Löbl, 1975

206C21A9-61B5-5EEE-AB7B-9742EDEC3FFF

Fig. 10

Bryaxis mahunkai Löbl, 1975: 117.
Bryaxis leechanyoungi Nomura & Lee, 1993: 27; syn. nov.

##### Material examined

**(*N* = 60, 34**♂♂, **26**♀♀) . 1♂ (CBNUIC, dried). “Korea: Chungbuk Prov., Mt. Songnisan, Beopjusa-ro, Songnisan-myeon, Boeun-gun, 20 Sep 2019, 36°32'55.6"N, 127°51'19.8"E, 476 m, flood debris, Y.-J. Choi, J.-W. Kang”. 2♂♂, 11♀♀ (CBNUIC, dried). “Korea: Gangwon Prov., Garakjae-ro, Hwachon-myeon, Hongcheon-gun, 5 May 2019, 37°46'26.0"N, 127°54'48.0"E, 240 m, sifting leaf litter near stream, J.-S. Park”. 1♂ (CBNUIC, dried). “Korea: Gangwon Prov., Jangjeon-gil, Jinbu-myeon, Pyeongchang-gun, 11 Mar 2019, 37°27'58.0"N, 128°32'18.4"E, 901 m, sifting leaf litter & dead wood debris & moss, J.-W. Kang”. 7♂♂, 7♀♀ (CBNUIC, dried). “Korea: Gyeongbuk Prov., Gowol-gil, Yeongyang-eup, Yeongyang-gun, 19 Mar 2019, 36°38'48.1"N, 129°09'18.7"E, 265 m, sifting leaf litter, Y.-J. Choi”. 2♂♂ (CBNUIC, dried). “Korea: Gyeongbuk Prov., Yongmunsa-gil, Yongmun-myeon, Yecheon-gun, 1 Jun 2019, 36°43'45.0"N, 128°22'14.0"E, 358 m, sifting leaf litter & soil near stream, U.-J. Byeon”. 7♂♂ (CBNUIC, dried). “Korea: Gyeonggi Prov., Mt. Bukhansan, Daeseomun-gil, Deogyang-gu, Goyang-si, 23 Aug 2019, 37°39'43.7"N, 126°59'11.2"E, 491 m, sifting leaf litter & soil & dead wood debris, Y.-J. Choi, T.-Y. Jang”. 4♂♂ (CBNUIC, dried). “Korea: Gyeonggi Prov., Mt. Yeoninsan, Yongchu-ro, Gapyeong-eup, Gapyeong-gun, 15 Apr 2019, 37°51'29.5"N, 127°28'01.0"E, 193 m, sifting leaf litter & moss near stream, J.-Y. Kang, J.-W. Kang”. 5♂♂, 3♀♀ (CBNUIC, dried). “Korea: Gangwon Prov., Hwanseon-ro, Singi-myeon, Samcheok-si, 23 Aug 2018, 37°20'22.6"N, 129°03'28.5"E, 172 m, sifting leaf litter near mountain stream, Y.-J. Choi”. 5♂♂, 5♀♀ (CBNUIC, dried). “Korea: Jeonnam Prov., Mt. Heukseoksan, Biseuran-gil, Gyegok-myeon, Haenam-gun, 18 May 2019, 34°40'44.9"N, 126°37'10.9"E, 160 m, sifting mushroom & leaf litter & plant root under rock in bamboo forest, S.-H. Choi, U.-J. Byeon”.

##### Remarks.

**Figure 10.**
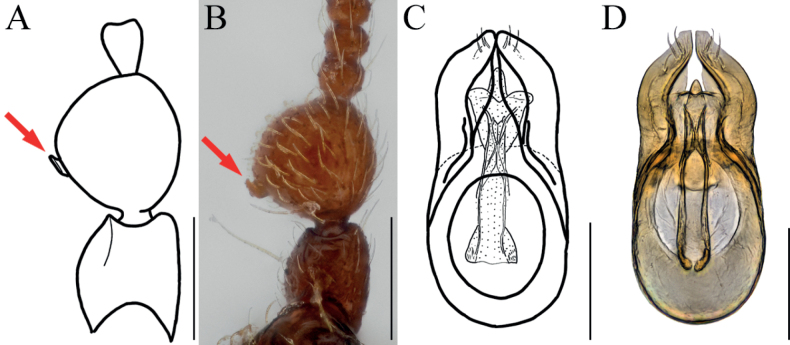
Diagnostic characters of *Bryaxismahunkai* Löbl. **A, B** antennal scapes and pedicels **C, D** aedeagi. Scale bars: 0.1 mm.

Adult males of this species are characterized by the following combination of characters: maxillary palpomeres II–III tubercular ventrally; antennal scapes tubiform, more curved on the internal side; pedicels globularly enlarged and a glandular nodule situated at the basal 1/3 (Fig. 10A, B, arrows); parameres with four setae each; and an endophallus consisting of two slender struts, converging subapically (Fig. 10C, D).

##### Comments.

Dorsal habitus of the holotype is available in Park and Jeon (2012; https://ecolibrary.me.go.kr/nibr/#/search/detail/5513253). Illustrations of antenna and aedeagus were obtained from Löbl (1975), and compared to those of specimens examined in this study. All specimens collected in Korea were recognized as *B.mahunkai* Löbl based on the antennal scapes (curved internally), pedicels (swollen and bearing upward glandular nodule), and the aedeagus (structure of endophallus).

##### Distribution.

Korea (Kaesong-si, Gyeonggi-do; Gapyeong-gun, Gyeonggi-do; Goyang-si, Gyeonggi-do; Boeun-gun, Chungcheongbuk-do; Hongcheon-gun, Gangwon-do; Samcheok-si, Gangwon-do; Pyeongchang-gun, Gangwon-do; Yeongyang-gun, Gyeongsangbuk-do; Yecheon-gun, Gyeongsangbuk-do; Haenam-gun, Jeollanam-do).

## ﻿Discussion

This study was the first revision of Korean *Bryaxis* since Nomura and Lee described eight new species in 1992–1993 [note that *Bryaxiscoreanus* Nomura & Lee, 1992 was subsequently synonymized with *Bryaxiskoltzei* (Reitter, 1887) (Nomura 1995)]. According to Kurbatov and Löbl (1995), subgenera *Arcobythus* Jeannel, 1958 and *Bythiniama* Jeannel, 1958 were synonymized with *Bryaxis* Kugelann due to the absence of informative characters to separate the genus into subgeneric groups. The adult males of *B.nemorosus* sp. nov. possess unadorned antennomeres and small eyes, which are thought to be linked to their shady habitat caused by the dense canopy. The features of this species are shown in cavernicolous species (e.g., elongated scapes and reduced eye sizes (Hlaváč 2006; Bekchiev and Hlaváč 2016). However, it is difficult to say whether it belongs to the same lineage as the other cavernicolous species, considering the isolated locality of *B.nemorosus* sp. nov.

**Figure 11.**
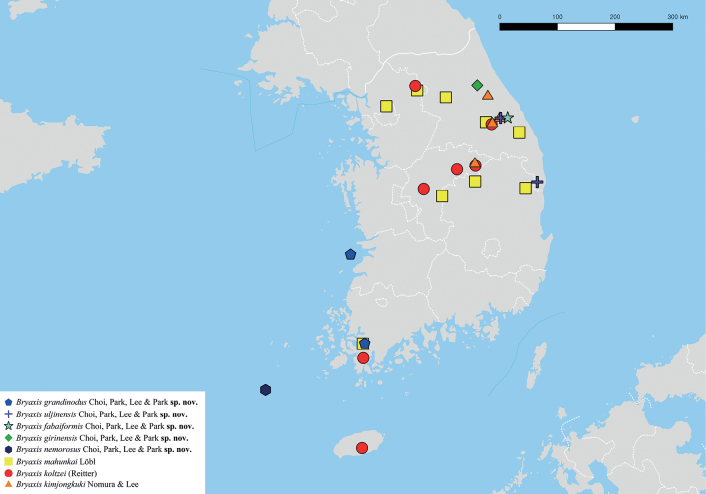
Collection localities. *Bryaxisgrandinodus* sp. nov. (blue pentagon); *B.uljinensis* sp. nov. (purple cross); *B.fabaiformis* sp. nov. (sky-blue star); *B.girinensis* sp. nov. (green diamond); *B.nemorosus* sp. nov. (navy hexagon); *B.mahunkai* Löbl (yellow square); *B.koltzei* (Reitter) (red circle); *B.kimjongkuki* Nomura & Lee (orange triangle).

This study added five new species based on 28 specimens. We were able to recollect only three of the species previously described. Of these, *B.koltzei* (Reitter) and *B.mahunkai* Löbl were very abundant over their ranges with hundreds of specimens collected. *Bryaxiskoltzei* is a very widespread species present throughout much of eastern Asia, from Korea, north to Russian Far East, and Japan, while *B.mahunkai* is endemic to Korea. *Bryaxiskimjongkuki* Nomura & Lee, also endemic to Korea was less abundant than these two, with about 50 specimens collected throughout its range. Two species, *B.grandinodus* sp. nov. and *B.uljinensis* sp. nov., were distributed in two localities each (Fig. 11), suggesting the potential for a wide habitat range.

## Supplementary Material

XML Treatment for Bryaxis

XML Treatment for Bryaxis grandinodus

XML Treatment for Bryaxis uljinensis

XML Treatment for Bryaxis fabaiformis

XML Treatment for Bryaxis girinensis

XML Treatment for Bryaxis nemorosus

XML Treatment for Bryaxis mahunkai

## ﻿Additional information

### Author ORCIDs

Yeon-Jae Choi https://orcid.org/0000-0003-0172-1128

Sun-Jae Park https://orcid.org/0000-0003-3759-6517

Seung-Gyu Lee https://orcid.org/0000-0001-5576-2799

Jong-Seok Park https://orcid.org/0000-0003-2063-2534

### Data availability

All of the data that support the findings of this study are available in the main text or Supplementary Information.
